# Optical Control of Tissue Regeneration through Photostimulation of Organic Semiconducting Nanoparticles

**DOI:** 10.1002/adhm.202200366

**Published:** 2022-07-28

**Authors:** Giada Onorato, Federica Fardella, Anna Lewinska, Federico Gobbo, Giuseppina Tommasini, Maciej Wnuk, Angela Tino, Maria Moros, Maria Rosa Antognazza, Claudia Tortiglione

**Affiliations:** ^1^ Istituto di Scienze Applicate e Sistemi Intelligenti “E. Caianiello” Consiglio Nazionale delle Ricerche Via Campi Flegrei 34 Pozzuoli 80078 Italy; ^2^ Department of Biotechnology Institute of Biology and Biotechnology Faculty of Biotechnology University of Rzeszow Pigonia 1 Rzeszow 35–310 Poland; ^3^ Center for Nano Science and Technology @PoliMi Istituto Italiano di Tecnologia Via Pascoli 70/3 Milano 20133 Italy; ^4^ Politecnico di Milano Dip. di Fisica P.zza L. Da Vinci 32 Milano 20133 Italy; ^5^ Department of Biology Faculty of Biotechnology University of Rzeszow Pigonia 1 Rzeszow 35–310 Poland; ^6^ Instituto de Nanociencia y Materiales de Aragón C/Mariano Esquillor 15 Zaragoza 50018 Spain; ^7^ Institute of Biosciences and Bioresources National Research Council Via Pietro Castellino 111 Napoli Italy; ^8^ Instituto de Nanociencia y Materiales de Aragón C/Mariano Esquillor 15 Zaragoza 50018 Spain

**Keywords:** light responsive conjugated polymers, tissue regeneration, model organisms, organic semiconductors, optical stimulation

## Abstract

Next generation bioengineering strives to identify crucial cues that trigger regeneration of damaged tissues, and to control the cells that execute these programs with biomaterials and devices. Molecular and biophysical mechanisms driving embryogenesis may inspire novel tools to reactivate developmental programs in situ. Here nanoparticles based on conjugated polymers are employed for optical control of regenerating tissues by using an animal with unlimited regenerative potential, the polyp *Hydra*, as in vivo model, and human keratinocytes as an in vitro model to investigate skin repair. By integrating animal, cellular, molecular, and biochemical approaches, nanoparticles based on poly‐3‐hexylthiophene (P3HT) are shown able to enhance regeneration kinetics, stem cell proliferation, and biomolecule oxidation levels. Opposite outputs are obtained with PCPDTBT‐NPs (Poly[2,6‐(4,4‐bis‐(2‐ethylhexyl)‐4*H*‐cyclopenta [2,1‐*b*;3,4‐*b*′] dithiophene)‐*alt*‐4,7(2,1,3‐benzothiadiazole)], causing a beneficial effect on *Hydra* regeneration but not on the migratory capability of keratinocytes. These results suggest that the artificial modulation of the redox potential in injured tissues may represent a powerful modality to control their regenerative potential. Importantly, the possibility to fine‐tuning materials’ photocatalytic efficiency may enable a biphasic modulation over a wide dynamic range, which can be exploited to augment the tissue regenerative capacity or inhibit the unlimited potential of cancerous cells in pathological contexts.

## Graphical abstract

The photoexcitation of conjugated polymer nanoparticles enhances the regeneration of an amputated animal, the freshwater polyp *Hydra*. The modulation of intracellular ROS levels through NP of different compositions represents a powerful modality to augment or inhibit the regenerative capacity of adult tissues for therapeutic purposes.

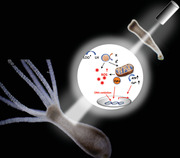

## 1 Introduction

Tissue repair/regeneration is one of the most fascinating biological capabilities of multicellular organisms. After an injury, various intracellular pathways and intercellular communication must be activated to establish a new tissue integrity and homeostasis. Often, when wounds or damage occur, stem cell compartment assumes a variety of regenerative decisions through which it controls the proliferation and differentiation until the cellular content of the tissue has been restored.^[^ ^1^ ^]^ Concomitantly, the transepithelial electrical potential is active by promoting cell migration from the wound edges. A measurable electric field persists until complete wound re‐epithelialization is achieved.^[^ ^2^ ^]^ Thus, studying the molecular mechanisms by which animals heal wounds or regenerate missing parts of the body can greatly improve our understanding of tissue patterning/organ regeneration in humans and might help to identify novel strategies to rescue the lost regenerative capacity. Several strategies are being proposed by bioengineers, spanning from reprogramming protocols inducing adult human tissues to regenerate whole organs, to the development of optimal culture conditions for growing and differentiating stem cells outside our bodies, to the fabrication of biomaterials injected to injury sites, recruiting endogenous stem cells in vivo, or enhancing tissue repair processes. Alongside biochemical and genetic networks, in the last decade bioelectrical signaling has gained an important role as a biophysical master regulator, controlling cell behaviors (proliferation, differentiation, migration) in both developmental and regeneration contexts.^[^ ^2^, ^3^ ^]^ For instance, robust evidence demonstrated the instructive role played by bioelectric signals during planaria regeneration, mirroring organ‐ and organism‐scale patterning processes described in vertebrate and invertebrate models.^[^ ^2^, ^3^ ^]^ These findings inspired recent approaches for the artificial modulation of bioelectrical signals, based on advanced materials and devices, acting through several possible mechanisms: i) capacitive stimulation, i.e., the generation of a localized, static electric field able to modulate the cell membrane equilibrium potential and capacitance, and ii) faradaic and electrochemical stimulation, i.e., the generation of a net electric current, with a possible concomitant increase of intracellular reactive oxygen species (ROS) concentration and subsequent oxidative stress signaling. ROS have complex influences on the cells, depending on their concentration. Indeed, beside their high reactivity linked to a disruption of macromolecules such as proteins, lipids, and DNA, recent research identified low level of ROS as important actors in most of the signaling cascades involved in cell development, proliferation, and survival, constituting important second messengers and being beneficial to metabolic activity.^[^ ^4^ ^]^ Thus, the capability to finely regulate ROS concentration holds a high therapeutic potential.

So far, however, despite recent advances in the understanding of the biology of healing, the rate of transformation from theory to a practical application has been slow, mainly due to i) the limited consideration of the use of smart functional materials: spare available reports mainly focused on low light level modulation techniques, but few examples exist on the use of smart functional materials as ROS generators for regenerative applications; ii) the lack of accurate wound model systems and the deficiency in the technological innovation of medical devices.

Among promising exogenous electroactive materials, semiconducting polymers deserve particular attention. Indeed, the synergistic use of visible light excitation and photo‐electrochemically active materials offers the opportunity to finely tune the concentration of ROS in a spatially and temporally selective manner, even at the subcellular level.

It has been recently reported that organic semiconductors sustain electrochemical reactions in a biological environment upon photoexcitation.^[^ ^5^ ^]^ Still, the use of conjugated polymers, and in particular of the prototypical photovoltaic material poly‐3‐hexylthiophene (P3HT) to optically promote tissue regeneration and wound healing has been explored only to a limited extent.^[^ ^6^ ^]^ Examples include P3HT/polycaprolactone (PCL) fibers, capable to increase cell proliferation and extracellular matrix secretion upon white light stimulation,^[^ ^7^ ^]^ P3HT micropillars, promoting an increase in the length of neurites and axons of embryonic cortical neurons as an effect of optical excitation,^[^ ^8^ ^]^ P3HT thin films, enhancing endothelial precursor proliferation and tube formation through optical modulation of intracellular Ca^2+^ signaling,^[^ ^9^ ^]^ fibrous membrane heterojunction composed of P3HT, PCL and polypyrrole (PPY), shown to facilitate neurogenesis after photostimulation of PC12 cells.^[^ ^10^ ^]^ In the reported examples, P3HT represented so far the material of choice, and in vitro cell cultures were used as models, investigating its proliferation and differentiation performance, while in vivo data are limited.^[^ ^11^ ^]^

Within a translational perspective, it is therefore of crucial importance to explore: i) other material possibilities among all those available in the broad portfolio of conjugated polymers, and ii) to use relevant animal models that go beyond in vitro cellular research.

In this scenario, we considered here two conjugated polymer nanoparticles (NPs), in water dispersions, based both on the reference material P3HT and on the red‐light absorbing (Poly[2,6‐(4,4‐bis‐(2‐ethylhexyl)‐4*H*‐cyclopenta [2,1‐*b*;3,4‐*b*′] dithiophene)‐*alt*‐4,7(2,1,3‐benzothiadiazole)] (PCPDTBT) polymer. Our choice was motivated by considering two fundamental requirements for the ideal material, besides photo‐electrochemical efficiency: i) capability to be injected as a water‐soluble dispersion that is stable in a physiological environment allowing spatial control the cell activity; ii) optical absorption spectrum in the red/NIR region, to facilitate optical excitation through the tissue and minimize light scattering effects, thus maximizing light penetrability.^[^ ^12^ ^]^ As an in vitro system, human primary keratinocytes were chosen as a model to investigate skin repair. Although in vivo repair is a complex process involving a multitude of other cell types (peripheral blood cells, resident skin cells, neurons) and regulatory molecules (extracellular matrix, cytokines, chemokines, growth factors), plus inflammatory and remodeling phases, the cell migration assay represents a robust laboratory technique to predict wound healing performance^[^ ^13^ ^]^.

As an in vivo model, we select *Hydra vulgaris* due to its remarkable regenerative potential. Among the simplest organisms that evolved during animal evolution, the freshwater polyp *Hydra vulgaris* is a powerful system to understand how complex anatomical structures emerge from the activity of individual cells.^[^ ^14^ ^]^ Shaped like a hollow tube a few millimeters long, with a foot to anchor to a substrate and a head surrounded by a tentacle crown, *Hydra* presents a tissue‐like organization, the whole body wall, from foot to tentacles, made of just two epitheliomuscular cell layers, the ectoderm facing the outer medium and the endoderm the inner cavity. The epitheliomuscular cells continuously divide, migrate toward the body extremities and together with stem cells interspersed in between the two layers ensure a constant cell turnover and differentiation process, drive asexual reproduction by budding, and maintain constant animal size. The high plasticity of the tissue underlies its ability to regenerate complete animals from amputated body parts, or from tiny pieces of excised tissue,^[^ ^15^ ^]^ thus representing a unique model to identify regulators or to validate new compounds to augment this process for therapeutic purposes. Our group succeeded in using *Hydra* as a versatile tissue‐like model to test interaction with a variety of nanostructured materials, from inorganic metal‐based nanoparticles (quantum dots, gold, and magnetic NP)^[^ ^16^ ^]^ to organic semiconducting compounds for bioelectronic purposes.^[^ ^17^ ^]^ Interestingly, we recently showed that living polyps incubated with a conjugated oligomer were able to form electronically conducting and electrochemically active µm‐sized domains fully integrated within *Hydra* tissue and the secreted mucus, suggesting the possibility of modulating biological functions in vivo through self‐organized electronics.^[^ ^17b^ ^]^ Furthermore, because *Hydra is a* body made of cells all electrically excitable and capable of generating and propagating electrical action potentials in response to exogenous stimulation,^[^ ^18^ ^]^ it is also a natural model system for studying the effect of bioelectrical signaling on morphogenesis, paralleling pioneering studies by M. Levin on planaria.^[^ ^2a^ ^]^ Recently, tunable external electric fields have been shown either halting or promoting *Hydra* regeneration, indicating a clear instructive role of the electric process also in *Hydra* morphogenesis.^[^ ^19^ ^]^ Finally, we recently show that P3HT‐NPs efficiently modulate *Hydra* photo‐behavior and induce overexpression of genes involved in the light transduction pathway.^[^ ^20^ ^]^

In the present work, we show that white light stimulation of P3HT and PCPDTBT NPs affects the regeneration efficiency of the two selected biological systems, in vivo and in vitro. In particular, conjugated polymer NPs modulate the *Hydra* regeneration and the migratory capabilities of keratinocytes in a bimodal way, either inhibiting or accelerating them, depending on the material of choice. Biochemical assays to estimate changes in the intracellular ROS levels show a direct effect of NP photostimulation on DNA and lipid oxidation, providing new opportunities for the controlled release of ROS for beneficial therapeutics in the field of tissue engineering. Nanotechnology tools based on NP dispersions, seamlessly integrated into living tissues, open the path to novel approaches for the optical modulation of various biological functions.

## 2 Results

### 2.1 Photostimulation of Organic Semiconducting NPs Enhance Regeneration In Vivo

**Figure 1:**
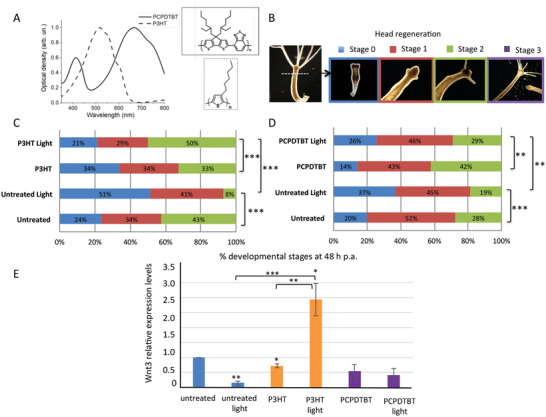
Photostimulation of semiconducting polymer NPs enhances *Hydra* head regeneration. A) Structure and spectral profile of the semiconducting polymers used in this study. B) Classification of developmental stages of regenerating heads, taken at 24 h intervals. C‐D) Impact of NP photostimulation on the regeneration efficiency of *Hydra*. Whole polyps either untreated or treated 24 h with 100 µg ml^–1^ P3HT‐NP (C) or PCPDTBT‐NP (D) were thoroughly washed, bisected and allowed to regenerate the head under white light (intensity 0.124 mW mm^–2^) or unilluminated (control animals). The percentage of regenerating heads (*n* = 30) were monitored every 24 h for their developmental stage. Distribution of developmental stages 24 h and 72 h p.a. are reported in Figure S3, Supporting Information. 3 independent biological replicates were performed (*n* = 90). Statistical comparisons were performed using the Chi‐square test and the Graphpad Prism 9 software (*P* values: * = *P* < 0.05; ** = *P* < 0.01; *** = *P* < 0.001), Tables S1 and S2, Supporting Information, reports *P* values for all comparisons. E) Relative expression levels of *Wnt3/EF1α*. 24 h p.a. Polyps treated as in C and D were processed for RNA extraction and qRT‐PCR. 2 biological replicates, each with 3 technical repeats were performed and statistical analysis was performed by unpaired Student t‐test.

The synthesis of light‐sensitive P3HT‐ and PCPDTBT‐NPs, and control, opto‐electrically inert NPs made of Poly(methyl methacrylate) (PMMA), is described in the Methods section. **Figure** **1**A shows the chemical structure and the optical absorption spectra of P3HT and PCPDTBT‐NPs aqueous dispersions, mainly absorbing in the green and in the red region of the visible light, respectively. Figure S1, Supporting Information, shows NPs size distribution and representative SEM images. Spherically shaped P3HT, PCPDTBT, and PMMA NPs have an average diameter of 230 ± 82 nm, 159.4 ± 63 nm, and 176.1 ± 48 nm, respectively. All dispersions displayed good stability over time, without showing aggregation effects over several weeks (data not shown). Zeta potential values for P3HT and PCPDTBT NPs are, respectively, −35 ± 8 mV, −2 ± 0.5 mV.

Polymer NPs were administered to groups of 30 polyps for 24 h to allow efficient internalization into animal tissues. The working concentration of P3HT‐NPs (100 µg mL^–1^) was selected on the base of extensive toxicity and biocompatibility evaluations previously performed, showing no adverse effect of P3HT‐NP on animal morphology and reproduction rate.^[^ ^20^ ^]^ For PCPDTBT‐NP a dose‐response toxicological assay was performed showing healthy morphology of polyps continuously incubated with the same dose (100 µg mL^–1^)(Figure S2B‐C, Supporting Information). A detailed morphometric analysis on whole polyps was performed also in presence of light stimulation to check for adverse effects played by photostimulated NPs in absence of any manipulation (Figure S2D‐E, Supporting Information). Animals were bisected and allowed to rebuild missing body parts over 72 h, the period normally required to completely differentiate new heads with tentacles. For the morphometric analysis to quantitatively estimate the kinetic of the regeneration under the diverse conditions a numerical value was assigned to each developmental stage: stage 0 at wound closure; stage 1 at beginning of tentacle morphogenesis (tentacles appear as buds); stage 2 when tentacles reach 2/3 of a mature tentacle length and stage 3 when the process is completed (Figure 1B). Optical stimulation of NPs‐treated stumps was accomplished by using a white light source (power density 0.124 mW mm^–2^), previously shown able to modulate the photo‐behavior of whole animals treated with P3HT‐NP,^[^ ^20^ ^]^ while the effect on amputated polyps has not been described to date. For this reason, the regeneration efficiency for each NP test was compared to control conditions, namely untreated/not irradiated (untreated), untreated/irradiated (untreated light), and treated/not irradiated (NP) (Figure 1C,D). In each photostimulation experiment, all control conditions were repeated simultaneously, to take into account the animal variability; moreover, the large number of animals (30 polyps/biological replicate, *n* = 90) enabled statistical analysis of the data. Data show a clear inhibitory effect on the regeneration efficiency played by the light irradiation alone: at the critical time point (48 h post amputation, p.a.) most of the illuminated, NPs untreated stumps (untreated light) are at stages 0 and 1, contrary to the condition of no light, with much higher percentages of stumps (43% versus 8% and 28% versus 19%, Figure 1C,D, respectively) presenting well‐developed tentacles and classifiable at stage 2. While a few attempts described the photosensitivity of *Hydra* to light of different wavelengths,^[^ ^21^ ^]^ our finding on the modulation of the regenerative process by light is pioneering this field opening to deeper investigations on the mechanism of light perception in this eyeless animal and the physiological implications in natural environments. Interestingly, P3HT‐NPs photostimulation markedly enhances the regeneration dynamic (Figure 1C), recovering the inhibitory effect of the light. The photostimulation of PCPDTBT‐NPs produces a similar trend in enhancing the regeneration efficiency and recovering the light inhibition (Figure 1D), although less pronounced and not strictly related to the irradiation.

At the two other time points of monitoring, 24 h and 72 h p.a., similar trends were obtained, with distribution of developmental stages shifted to the corresponding time post‐amputation, i.e., almost all polyps at stage 0 at 24 h p.a. and at stage 2 at 72 h (Figure S3, Supporting Information). Similar experiments were carried out by treating *Hydra* polyps with electrically inert and light insensitive PMMA‐NPs, characterized by comparable dimensions (Figure S1, Supporting Information) and similar outcomes of the toxicological assay at the same doses (Figure S4, Supporting Information). Of note, no sizable effect on *Hydra* regeneration was detected in this case, thus confirming the role of the semiconducting polymer in the light‐mediated bioactivity (Figure S5, Supporting Information). Next, we performed a regeneration assay at a lower light intensity (0.06 mW mm^–2^). As expected, the inhibitory effect played by the light on the regeneration was abolished, while the different behavior of the two NPs clearly emerged, as the P3HT‐NP but not PCPDTBT‐NP significantly modulates the regeneration dynamic (Figure S6, Supporting Information).

To further dissect the response of the regenerating animals to the electroactive NPs the expression profile of *Wnt3*, a well‐characterized gene involved in the head regeneration process^[^ ^22^ ^]^ was investigated by real time quantitative reverse Transcription PCR (qRT‐PCR), a technique allowing to measure the mRNA levels of a specific gene, relative to an internal control.^[^ ^23^ ^]^ The graph of Figure 1E shows a significant enhancement of *Wnt3* transcript levels in P3HT‐NPs treated and illuminated polyps, compared to untreated animals, suggesting an active role of *Wnt*/*β*−catenin signaling cascade in the P3HT‐mediated photoinduced regeneration. Accordingly, the decreased *Wnt3* levels in PCPDTBT treated polyps may explain the absence of a robust enhancing effect on *Hydra* regeneration.

In addition to the pre‐patterning events, *Hydra* regeneration relies on stemness of epitheliomuscular and interstitial cells continuously undergoing self‐renewal by mitotic division and differentiation of head‐ or foot‐specific epithelial cells. While re‐organization of tissues at the wound site can occur without cell division (“morphallaxis”),^[^ ^24^ ^]^ head removal results in an immediate block and later up‐regulation of mitosis.^[^ ^25^ ^]^ This prompted us to estimate the effect of NP on cell proliferation rates in regenerates. Animals were dissociated into a suspension of fixed single cells maintaining their morphology (a process named maceration),^[^ ^26^ ^]^ allowing us to analyze the distribution of the different cell types and to quantify their relative abundance, which is unique of a physiological condition and indicative of the dynamic of proliferation and differentiation processes.^[^ ^25^ ^]^ Fast cycling interstitial stem cells (single and pairs of stem cells, named 1s + 2 s) normally complete a cell cycle in 24 h, while epithelial cells are slow cycling, complete a cell cycle in 48–72 h and represent 30% of total cells. To estimate the proliferation rate of stem and epithelial cells a Bromodeoxyuridine (BrdU) assay was performed, based on the capability of this Thymidine analog to be incorporated into DNA in place of Thymidine. Therefore, BrdU labels the cells that are undergoing or have recently undergone DNA replication, and it is used to estimate cell proliferation.

**Figure 2:**
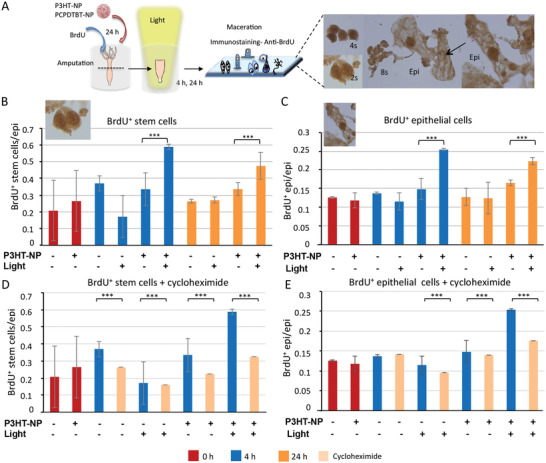
Photostimulation of P3HT‐NP augments cell proliferation rates during regeneration. A) Scheme of the experimental procedure. *Hydra* polyps were treated with P3HT‐NPs, then incubated with BrdU, and after washing amputated and allowed to regenerate missing heads (illuminated or not illuminated). At 0 h, 4 h and 24 h p.a. polyps were macerated and immunostained with anti‐BrdU antibody to visualize proliferating cells. On the right panel images of the different cell types are shown, recognizable by their morphology, i.e., stem cells are present as single or couple of cells (2 s), proliferating nematoblasts are present as nests of 4–16 cells (4 s, 8 s, and 16 s), epithelial cells include ectoderm and endodermal cells. BrdU+ cells show an intense brown colored nucleus, while negative cells do not (arrow). Relative amount of BrdU^+^ stem cells B) and epithelial (epi) cells C) prepared from treated polyps are indicated as red, blue and orange bars, corresponding at 0 h, 4 h and 24 h p.a. −/− = untreated/no light; +/− = treated/no light; −/+ = untreated/light; +/+ = treated/light. D‐E) Effect of cycloheximide on stem and epithelial cell proliferation. Polyps were treated with P3HT‐NP for 24 h, then with BrdU and finally with cycloheximide 50 µg mL^−1^ 15 min before cutting and 2 h after cutting. Red color indicates polyps at 0 min p.a.; blue, polyps at 4 h p.a. without cycloheximide; light orange, polyps at 4 h p.a. treated with cycloheximide. The graphs reporting the stem cells D) and epithelial E) proliferation rates clearly show for each experimental condition that the cycloheximide treatment (light orange bar) reduce the amount of BrdU+ cells relative to the corresponding rate in absence of cycloheximide treatment (blue bars). For each graph data are presented as means ± SD of three biological replicates (*n* = 600 cells). Statistical comparison among different conditions were analyzed by Student's *t*‐test using GraphPad Prism 9. *P*‐values of less than 0.05 were considered significant: *P < 0.05; **P < 0.01; ***P < 0.001.

Whole animals were treated with P3HT‐NP and incubated with BrdU, bisected, and macerated immediately, 4 h, and 24 h p.a. (**Figure** **2**A). Dividing cells, in phase S of the cell cycle, were identified by immunoreaction with anti BrdU antibody. Histograms in Figure 2B show a significant enhancement of the BrdU^+^ stem cells in presence of P3HT NPs and light irradiation, at both 4 h and 24 h time points (blue and yellow data, respectively). The boost in the cell proliferation rate (doubling as compared to the time zero) was greater in stem cells compared to epithelial cells (Figure 2C), which agrees with the slower cell cycling activity of the latest ones. Of note, the inhibitory action played by the light on stem cell proliferation at 4 h p.a. well correlates with the in vivo delay of regenerating heads under light exposure (Figure 1) while the enhanced proliferation of epithelial and stem cells is in line with the faster regeneration dynamic induced by NP photostimulation.

To further investigate whether the inhibition of the cell proliferation played by the light illumination was dependent upon the cell cycle arrest, the cycloheximide protein inhibitor, known to prevent cell mitosis, was used after BrdU treatment. Figure 2D‐E, comparing the BrdU^+^ cell amounts obtained in the absence and presence of cycloheximide, shows that the division of both slow and fast cycling cells in regenerating animals, 4 h p.a., was prevented. Moreover, the enhancing effect of P3HT‐NP photostimulation on cell proliferation was abolished, thus indicating a possible effect on phase M/G1 transition. Therefore, P3HT NPs photostimulation, besides modulating gene expression, also boosts the proliferation of fast cycling cells which, in turn, causes the faster differentiation of head‐specific structures. Overall, the trend observed for the cell proliferation rates mirrors the in vivo data, suggesting a clear modulation of the NP photostimulation on the dynamic of head regeneration. Remarkably, in the case of PCPDTBT NPs, the proliferation rate of stem cells was only slightly enhanced 4 h and 24 h p.a. (Figure S7, Supporting Information), while not significant effect was detected on epithelial cells, in agreement with the less pronounced effect observed in vivo for PCPDTBT‐NP treated regenerants.

### 2.2 NP Photostimulation Induce Changes of Intracellular Redox Equilibrium In Vivo

**Figure 3:**
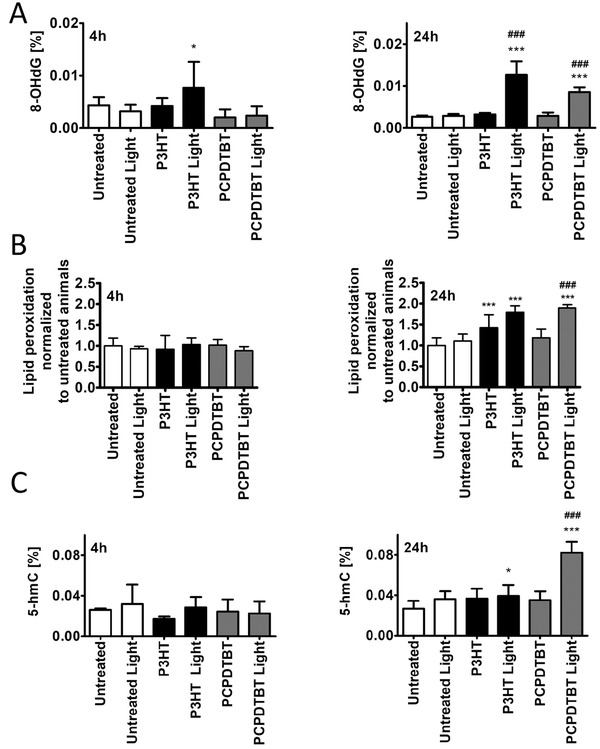
Photostimulation of semiconducting NPs modulates intracellular redox milieu. A) Time dependent changes in 8‐OHdG levels in polyps treated with P3HT‐NP (black bars) and PCPDTBT‐NP (grey bars) and exposed to led white light for 4 h (left) or 24 h (right). For each NP, the 8‐OHdG levels were compared to untreated and untreated/light, and NP treated conditions. B) Lipid peroxidation was measured in polyps treated as above for 4 h (left) and 24 h (right). C) 5‐hmC levels measured in polyps treated as above, for 4 h (left) or 24 h (right). Bars indicate SD, *n* = 6, ***P < 0.001, *P < 0.05 compared to untreated animals (ANOVA and Dunnett's a posteriori test). Two‐tailed paired Student's *t*‐test was also used to compare NPs treated animals to NPs/light illuminated animals; ^###^ *P* < 0.001.

The photocatalytic activity of P3HT thin films has been recently reported to underlie optical modulation of intracellular ROS and Ca^2+^ concentration in in vitro cell cultures.^[^ ^5^, ^9^ ^]^ P3HT‐NPs exhibit photoelectrochemical processes that lead to oxygen reduction and subsequent production of ROS,^[^ ^27^ ^]^ as well as modulation of the antioxidant capacity in *Hydra*.^[^ ^28^ ^]^ Based on previous evidence, we tested by additional biochemical methods the oxidation of other intracellular biomolecules, i.e., lipids and DNA, possibly induced by photoexcitation. We measured i) the levels of 8‐hydroxy‐2‐deoxyguanosine (8‐OHdG), one of the predominant forms of free radical‐induced DNA oxidative lesions, widely used as a biomarker for oxidative stress and carcinogenesis ^[^ ^29^ ^]^ and ii) the lipid peroxidation, a process under which free radicals attack lipids containing carbon‐carbon double bonds, especially polyunsaturated fatty acid, thus the end products of this chain of reactions (i.e., malondialdehyde) are used as markers of oxidative stress.^[^ ^30^ ^]^ Intact polyps were exposed for different periods to the same light density used for regeneration experiments and then processed for oxidative stress‐based DNA and lipid modifications (**Figure** **3**). Intact polyps instead of regenerating stumps were used, as the boost of ROS physiologically produced by the amputation would hinder data interpretation. Light‐mediated effects were not observed in untreated animals (Figure 3), while, in presence of NPs, the photostimulation resulted in elevated levels of 8‐OHdG. The effects were more pronounced after 24 h illumination, as shown by statistically significant differences in 8‐OHdG levels detected between animals treated with PCPDTBT or P3HT NPs and exposed to light, as compared to the unilluminated samples (Figure 3A). A 4 h‐long stimulation did not affect lipid peroxidation under any condition (Figure 3B) while a 24 h‐long photostimulation of both NPs increased lipid peroxidation levels (Figure 3B), which was also detected in case of P3HT‐NP in absence of irradiation. Finally, 5‐hydroxymethylcytosine (5‐hmC) levels were evaluated. 5‐hmC, an oxidized derivative of 5‐methylcytosine (5‐mC), represents a well‐characterized epigenetic mark of development, aging, cancer, and neurodegenerative disorders, positively correlating with gene transcript levels.^[^ ^31^ ^]^ In addition, 5‐hmC levels increase during the stress response^[^ ^32^ ^]^ and we have recently shown their modification by some antioxidants.^[^ ^33^ ^]^ No differences were detected at 4 h (Figure 3C) while 24 h photostimulation resulted in elevated 5‐hmC levels compared to untreated animals (Figure 3C), the effect of PCPDTBT‐NP being more evident than that of P3HT‐NP. Together, these results suggest that after prolonged NP photostimulation, an oxidative stress‐mediated response can be induced in *Hydra*, causing a redox imbalance and in turn a moderate oxidation of DNA and lipids (increased 8‐OHdG, lipid peroxidation, and 5‐hmC levels). This mild oxidative stress can impact on cell metabolism, promote wound healing, and prevent cell death and tissue damage similarly to the effects produced by low‐level light therapy.^[^ ^34^ ^]^ It is widely accepted that a stress‐mediated hormetic response (i.e., a biphasic dose response characterized by stimulation or beneficial effect at low dose and by an inhibitory or toxic effect at high dose) can modulate cell proliferation, wound healing, and angiogenesis.^[^ ^35^ ^]^ According to a hormetic response, the mild increase in 5‐hmC levels induced by P3HT‐NP may promote head regeneration and cell proliferation (Figures 1C, and 2B,C), while elevated levels of 5‐hmC induced by PCPDTBT‐NP may account for the limited regenerative capacity (Figure 1D). Moreover, a slight increase in the levels of 5‐hmC may also result in hypomethylation‐mediated induction of gene expression. For example, the conversion of 5‐mC to 5‐hmC is considered to be an important regulatory mechanism for the expression of *Wnt* genes.^[^ ^36^ ^]^ Consequently, increased levels of *Wnt3* mRNA levels were observed in *Hydra* after photostimulation with P3HT‐NP photostimulation (Figure 1E). Therefore, we postulate that 5‐hmC may play an important role during NP photostimulation‐mediated hormetic response.

### 2.3 Bimodal Effect of NP Photostimulation on Keratinocyte Migratory Capacity

**Figure 4:**
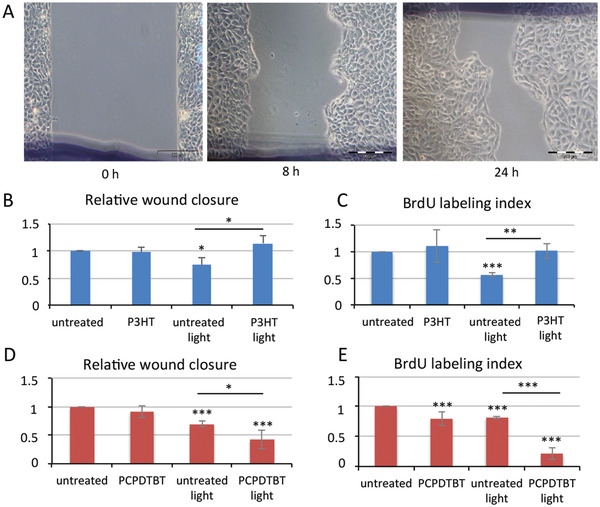
Photostimulation of semiconducting NPs modulates keratinocyte migration. A) HaCaT cells either treated with NP or untreated were seeded into cell culture dishes. At time zero the insert was removed, and cells were allowed to close the gap either at light density of 0.124 mW mm^–2^ or at ambient light. Scale bars 200 µm B) Quantification of wound closure, estimated as cell‐covered area relative to the initial wound area, for each condition, was determined at 24 h after wounding. Experiments were performed in triplicate. C) BrdU labeling index (%). For each condition cells were pulsed 4 h with BrdU and immunostained with anti BrdU antibody. D) Relative wound closure and E) BrdU labeling index for HaCaT cells treated with PCPDTBT‐NP. Data represent the average ±SD of three independent experiments. Statistical comparisons were performed using unpaired Student *t*‐test; *P < 0.05; **P < 0.01; ***P < 0.001.

With the general aim to evaluate whether the NP photostimulation could more generally enhance the regenerative potential in vertebrates, we used immortalized human keratinocytes (HaCaT cells) as in vitro system to monitor collective cell migration, a hallmark of wound repair. In cutaneous wound healing, skin cells migrate from the wound edges into the wound to restore skin integrity. Analysis of cell migration in vitro may allow to quantify alterations in cell migratory capacity in response to experimental manipulations.^[^ ^13^ ^]^ HaCaT cells were treated 24 h with NPs 100 µg mL^–1^ and seeded on appropriate cell culture dish enabling the creation of an artificial gap on a confluent cell monolayer. Irradiation was performed with white led light at same intensity used in *Hydra* (0.124 mW mm^–2^) and 24 h later the cell‐covered area was calculated and compared to treated cells not irradiated and to corresponding controls (untreated cells, either exposed to light or not). **Figure** **4**A shows representative images of wound edges migrating to progressively close the gap at the time 0 h, 8 h, and 24 h after wounding. The wound closure in irradiated cells was reduced compared to the not irradiated cells (Figure 4B) and correlated to a significant inhibition of the cell proliferation played by optical excitation by itself, as also indicated by the reduced BrdU labeling index in the same condition (Figure 4C). Remarkably, P3HT‐NPs treatment fully prevented this inhibition, indicating a clear effect of the P3HT‐NP photostimulation on keratinocyte migration and proliferation. Conversely, control PMMA‐NPs showed no effect on wound closure (Figure S8, Supporting Information). At variance with P3HT‐NP effect, PCPDTBT‐NP photostimulation caused a strong inhibition of the wound closure (Figure 4D), in addition to the effect of the light alone. BrdU assay shown in Figure 4E showed also a strong inhibition of the cell proliferation under illumination, indicating a possible mechanism preventing the wound healing. Overall, data reported in Figure 4 confirm the different behavior of P3HT and PCPDTBT‐NPs to modulate the migrating and proliferating capabilities of keratinocytes, mirroring data achieved in *Hydra* head regeneration (Figure 1) and cell proliferation (Figure 2).

## 3 Discussion and Conclusions

The possibility to control biological function using intracellular light actuators has several advantages compared to other traditional approaches that rely on the light sensitivity of cells and thus requiring genetic engineering to confer optical responsiveness to the desired cell type. The availability of a nanosized device, fully integrated with the tissue and enabling optical control of the cell functions, may overcome not only cost and time‐consuming genetic approaches, but also the issue of spatial and temporal resolution, invasiveness, stiffness, and low compliance of the planar light actuators. Nanoparticles based on organic semiconductors, such as P3HT and PCPDTBT represent valid alternatives, and were shown in diverse biological systems to transduce the light stimulus and evoke a cellular response or animal behavior.^[^ ^11^, ^20^, ^27^ ^]^ P3HT‐NPs when directly injected into the eye of a rat model of retinisis pigmentosa were shown to functionally replace damaged photoreceptors and restore visual acuity with high spatial resolution, leading to the transformative concept of liquid prosthesis.^[^ ^11^, ^37^ ^]^ Here, we exploited the application potential of polymer NPs by targeting optical modulation of the tissue regeneration. We addressed this aim by using two in vivo and in vitro regenerative models. Photostimulation of P3HT‐NPs caused an acceleration of the regenerative process in *Hydra* sustained by a strong enhancement of cell proliferation rates and *Wnt3* gene expression and promoted the cell migratory capabilities of keratinocytes in a two‐dimensional monolayer. PCPDTBT‐NPs behave differently, acting to a minor extent on *Hydra* regeneration, while causing a strong inhibition of keratinocyte migration and proliferation capabilities. Future testing of both semiconducting NPs in 3D models of skin may help to understand the effect of light‐activated NPs on fibroblasts and extracellular matrix properties, filling the gap between 2D monolayer and more clinically relevant in vivo models of wounds.

A detailed understanding of physical/chemical mechanisms occurring at the interface between the polymer NPs surface and the cellular cytosol or the in vivo tissue is not straightforward. Their optoelectronic properties were extensively characterized in a physiological‐like environment,^[^ ^38^ ^]^ and it was reported that both materials preserve the capability to generate charges upon photoexcitation, while maintaining good stability properties.^[^ ^39^ ^]^ However, two important differences between the two polymer NPs should be taken into account. First, P3HT‐NPs were realized without the use of surfactants, while PCPDTBT‐NPs were synthesized by making use of Polyvinyl alcohol (PVA). This different fabrication process, together with different average diameters, can lead to a different chemical/physical interaction with the biological environment, either the cell cytosol or the animal tissue, and may account for a different internalization rate. Furthermore, the zeta potential is strongly affected by the presence of the PVA surfactant, approaching in this case values close to zero, as opposed to the negative values around ‐35 mV typical of P3HT‐NPs. A second important difference between P3HT‐NPs and PCPDTBT‐NPs consists of the different energetic levels of the materials. The PCPDTBT polymer is intrinsically characterized by a higher photon conversion efficiency, even when used in pristine form without an electron acceptor material.^[^ ^40^ ^]^ Interestingly to this work, both P3HT and PCPDTBT display photocatalytic activity, and have been reported to trigger photoelectrochemical reactions in an aqueous environment.^[^ ^41^ ^]^ Interestingly, the PCPDTBT LUMO level is more favorable to oxygen reduction relative to P3HT (Figure S9). This may account for more efficient ROS generation under similar photoexcitation conditions, thus explaining the differences observed in the cell behavior, even in the case all other conditions (internalization capability, chemical/physical interaction with the cell environment, and efficiency of charge generation and recombination processes) are equal.

More precisely, the cell responses to the NP photostimulation may reflect a hormetic behavior, i.e., a biphasic dose response characterized by a stimulation or beneficial effect at low dose and by an inhibitory or toxic effect at high dose. Recently, we have shown that nanodiamonds and silica nanoparticles at low concentrations promote a hormetic response in human skin fibroblasts in vitro. In fact, at low doses, they exert mild stress‐induced beneficial effects through improved survival, wound healing capacity, longevity, repair, and function of human cells,^[^ ^42^ ^]^ while at high doses they are toxic.

Although the mechanism underlying the NP‐mediated translation of the light stimulus into modulation of metabolic pathways is not deciphered yet, we do show significant changes in the redox balance induced by the two semiconducting NPs, which may positively or negatively affect cell proliferation, depending on the biological model and on the cell physiological state. Interestingly, inhibition of the cell proliferation by PCPDTBT‐NP might find obvious applications in all therapeutic purposes where inhibition of cell migration and proliferation is demanded, i.e., anticancer strategies.

**Figure 5:**
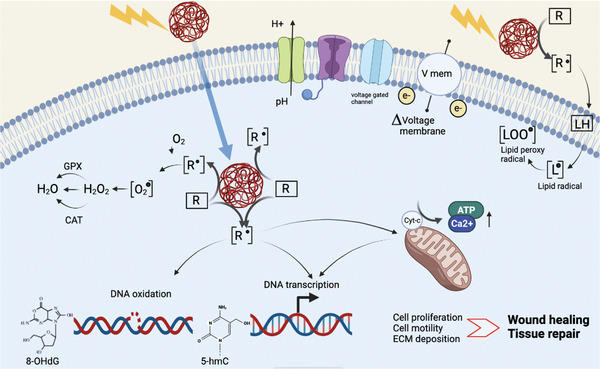
Biological pathways possibly induced by photostimulation of semiconducting polymer NPs. Photoelectrochemical reactions possibly occurring at the NP surface (shown as red balls) may induce free radical formation (shown as black dot) on different biomolecules (R), or lipids (LH), present in their neutral form (framed by a rectangle) in the cell cytoplasm (light blue background) or in the extracellular aqueous medium (beige background). As effect of photostimulation, highly reactive radical species may be produced (identified by square brackets) and depending on the subcellular localization they may induce several effects, such as 1) biomolecule oxidation: membrane lipids can generate lipid and lipid peroxy radicals; DNA oxidative lesions induced by free radicals may increase 8‐hydroxy‐2‐deoxyguanosine (8‐OHdG) levels 2) increased transcription of genes involved in cell proliferation (i.e *Wnt3*), motility, extracellular matrix (ECM) deposition 3) increased 5‐hmC levels, an oxidized derivative of 5‐methylcytosine (5‐mC), positively correlating with gene transcription during the stress response 4) oxidative reactions at the mitochondrial interface, modulating ATP and Calcium levels. In addition 5) changes in the voltage membrane potential through voltage‐gated ion channels, H^+^ pumps, voltage sensitive small molecule transporters, represent crucial bioelectrical signals able to modulate morphogenetic processes.

The possible biological pathways activated by NP photostimulation are schematically described in **Figure** **5**. The most probable photoexcitation mechanism may be sustained by photoelectrochemical reactions occurring at the NP surface and taking place either extracellularly or the following internalization into the cell. ROS plays a pivotal role in the orchestration of the normal wound‐healing response, acting as secondary messenger‐signaling molecules.^[^ ^43^ ^]^ Increased cytoplasmic ROS concentration can result in several outcomes, from oxidation of membrane lipids or DNA, to the induction of detoxifying enzymatic reactions to restore physiological levels, and the activation of transcription factors, driving gene transcription.^[^ ^44^ ^]^ For example, ROS are necessary for the activation of the Jun N‐terminal kinase (JNK) and p38 mitogen‐activated protein kinases (MAPK) signaling pathways, which are known to facilitate the recovery of lost tissues.^[^ ^45^ ^]^ ROS levels can be modulated through a variety of sources, i.e., cellular respiration and metabolic processes. Thus in the proximity of mitochondria, respiratory chain redox reactions can be activated by NP photostimulation, leading to increased levels of ATP and Ca^2+^, as reported in the case of HEK cells and endothelial precursors,^[^ ^5^, ^27^ ^]^ and modulation of target gene expression, which may drive several phenomena, ultimately resulting in tissue repair or wound healing. In *Hydra* several components of the wound response have been identified by proteomic and transcriptomic analyses,^[^ ^22^, ^46^ ^]^ and includes Ca^2+^, ROS, MAPK signaling, apoptosis, and the activation of *Wnt* signaling.^[^ ^47^ ^]^ NP phostimulation may alter the ROS levels, and positively influence wound healing in presence of low ROS levels, or negatively by excessive ROS.

Furthermore, we do not exclude that NP photoexcitation may cause a modification of endogenous electrical signals.^[^ ^2^, ^3^ ^]^ Bioelectric signaling plays a central role in the coordination of cell behavior (differentiation, migration, proliferation) of a large number of cells via ion channels, pumps, and electrical synapses (gap junctions).^[^ ^3^, ^48^ ^]^ Ion transporters, for instance, triggered by photoexcitation may generate pH and voltage gradients, as well as ion fluxes, and the resulting electric field carrying instructive information may affect the wound healing both in *Hydra* and in keratinocyte models.

In a wide range of cells from yeast to human stem cells changes in the resting potential (*V*_mem_) have been shown affecting cell behaviors^[^ ^49^ ^]^ and mechanisms of endogenous bioelectricity in wound healing and regeneration have been recently reviewed.^[^ ^3^, ^50^ ^]^ It has been shown that on a wound hedge a transepithelial electrical potential promotes the cell migration from the wound edges.^[^ ^2a–c^ ^]^ The beneficial effect of this endogenous electrical field in guiding cell migration and nerve sprouting directly toward the wound edge has been exploited for therapeutic purposes. Pulsed electrical stimulation accelerates the healing rate of chronic dermal ulcers in human subjects,^[^ ^2^, ^51^ ^]^ enhances dermal fibroblast activity and myofibroblast transdifferentiation,^[^ ^52^ ^]^ promote cardiomyogenic and angiogenic differentiation^[^ ^53^ ^]^ of human embryonic stem cells. Thus, changes in the voltage membrane potential induced by the photostimulation of conjugated polymer nanoparticles may act as a bioelectrical signal, modulating in *Hydra* morphogenetic processes and in keratinocytes their coordinated migration at the wound hedge. The use of voltage sensitive dyes or the direct measurement of the membrane electrical properties represents interesting approaches to test this possibility.

In conclusion, our results open the way to the artificial modulation of biophysical signals in somatic tissues as powerful modalities to augment the regenerative capacity of adult tissues or inhibit the unlimited potential of cancerous cells in pathological contexts. Moving forward in clinical application, we can envisage regenerative strategies based on direct injection at the wound site, or the functionalization of hydrogels and wound dressings with these electroactive nanoparticles acting by modulating bioelectric signaling and promoting coordinated behaviors of cells at the wound site. Harnessing bioelectrical networks is a key step toward the ability to induce complex structures to be grown on‐demand, as required for transformative applications in regenerative medicine. On the other side, further improvement of material synthesis might enable photostimulation with low optical density, deeper tissue penetration, and long tissue persistence to perform systemic photostimulations.

A more in‐depth experimental characterization of the materials properties, taking into account all possible variables (just to cite some: hydrophobicity, morphology, chemical surface arrangement, molecular order, intrinsic conductivity, charge generation efficiency, internalization rate, NPs size, and zeta potential, role of surfactants) will allow to precisely design even more effective interfaces with the biological counterpart, thus finely tuning the overall efficacy of the proposed approach according to the application of interest. Overall, the concrete capability to influence in a desired, quantitative, and remotely controllable way the activity of stem and differentiated cells through transduction of optoelectronic stimulation may provide unique advancement to this field.

## 4 Experimental Section

### P3HT and PCPDTBT NPs Synthesis and Characterization

P3HT NPs were prepared by the reprecipitation method, in absence of surfactants, from commercially available polymer (Sigma Aldrich, molecular weight 20 000—45 000), used without further purification. The polymer was dissolved in tetrahydrofuran, and the solution was added to distilled water (solvent/nonsolvent volume in a 1:20 ratio).

PCPDTBT (1‐Material, molecular weight 40 000—50 000) and PMMA (Sigma‐Aldrich, molecular weight around 120 000) NPs were prepared by the miniemulsion method, from the commercially available polymers, and using Polyvinyl alcohol (PVA, Sigma Aldrich) as a surfactant. The polymer powders were dissolved in Chloroform (3 mg ml^–1^) and the solution was added to 5 mL of distilled water containing PVA (0.5%, in weight). The resulting emulsion was first sonicated in a tip sonicator (10 min, pulse mode: 40 s on 20 s off), then magnetically stirred for 2 h at 40 °C and finally for 12 h at room temperature, to guarantee complete removal of the organic solvent.

The dispersions were kept at room temperature and sonicated for 10 min before the characterization experiments or the administration to the samples.

Ultraviolet (UV)–visible (vis) optical absorption spectra were measured by using a PerkinElmer Lambda 1050 UV/Vis/NIR spectrophotometer. Dynamic Light Scattering (DLS) and zeta potential measurements were performed by means of a Malvern Zetasizer Nano ZS, by using 633 nm He‐Ne laser line excitation and collecting the scattered light at 173° angle with respect to the excitation beam.

All measurements were performed at 25 °C. Scanning electron microscopy images were acquired with a Tescan MIRA3.

### *Hydra* vulgaris Culturing

*Hydra vulgaris* (Cnidaria, Hydrozoa) was an invertebrate organism, which culturing and handling in laboratory did not require ethical approval from national authority.

*H. vulgaris* was cultured asexually in Hydra medium comprising 1 mM calcium chloride and 0.1 mM sodium hydrogen carbonate at pH 7. The animals were kept at 18 °C and fed on alternate day with freshly hatched *Artemia salina* nauplii with a 12:12 h light: dark regime. For all experiments, homogeneous population samples of *Hydra* were selected from adult polyps without buds, 24 h after their last feeding.

### Animal Treatment and Regeneration Assay

Groups of 35 *Hydra* were placed into different wells of a multiwell plate (24 wells) and incubated with P3HT‐NPs or PCPDTBT‐NP (100 µg mL^–1^) in *Hydra* solution in a final volume of 500 µL. After washing, using a scalpel, a sub‐hypostomal cut (80% body length) was performed, leading to polyp decapitation. Photostimulation was accomplished using a light emitting diode (LED) white light source (Edmund Optics, the power density of 0.124 mW mm^–2^, or 0.06 mW mm^–2^), and animals exposed 8 h d^−1^. Regenerating stages were inspected by stereomicroscope (SZX7, Olympus) and quantified 24, 48, and 72 h post‐amputation (p.a.). The experimental conditions to estimate the efficiency of regeneration in each experiment were untreated, NP/treated, untreated/illuminated, NP‐treated/illuminated.

### Gene Expression Analysis

Differences in gene expression profiles induced by NPs and/or light were assessed by quantitative reverse transcription polymerase chain reaction (qRT‐PCR).^[^ ^23^ ^]^ For each experimental condition, RNA was extracted from groups of 25 animals by purification in Trizol Reagent (Life Technologies) according to the manufacturer's instructions. RNA was quantified and quality checked by SmartSpec plus spectrophotometer (Biorad, Hercules, CA) and agarose gel electrophoresis, respectively. RNA samples were treated with DNaseI (Amplification grade, Invitrogen) according to supplier's instructions. The first‐strand cDNA was synthesized by a High Capacity cDNA Reverse Transcription Kit (Applied Biosystem) using 0.5 µg of DNA‐free RNA in a final volume of 10 µL. qRT‐PCR was performed in 10 µL of reaction mixture consisting of 1× Express Sybr Green (Invitrogen), serial cDNA dilutions, and 0.5 µM each primer. The reactions were processed using the StepOne Real‐Time PCR System (Applied Biosystem) according to the following thermal profile: 50 °C for 2 min, one cycle for cDNA denaturation (94 °C for 2 min), followed by 40 amplification cycles (94 °C 2 sec, 60 °C, 30 s). Specific pairs of primers were designed for each gene using the Primer3 program (http://frodo.wi.mit.edu). The expression profiles were analyzed by applying the ΔΔ*C*t method where the values of the gene of interest (*Wnt3*) were normalized to the values of the reference control gene (*Ef1α*).^[^ ^54^ ^]^

### *Hydra* Cell Proliferation Assays

Groups of 45 *Hydra* were placed into multiwells and treated for 4 h with P3HT‐NPs or PCPDTBT‐NP, and either exposed to led white light or kept at light ambient. 5‐Bromo‐2’‐Deoxyuridine BioUltra (*Sigma‐Aldrich*) 5 mM in *Hydra* solution was incubated with the polyps overnight. Furthermore, groups of 15 *Hydra* were treated with 50 µg mL^–1^ cycloheximide (cell cycle inhibitor in G2 phase) for 15 min before cutting. After cutting some *Hydra* were further treated with 50 µg mL^–1^ cycloheximide for 2 h during regeneration. As a control condition, both untreated animals and treated animals not illuminated were used. To estimate the effect of NP photostimulation on the cell proliferation rate, polyps were first dissociated into single cells and then BrdU^+^ cells were detected by immunocytochemistry. Briefly, single cell suspensions were obtained from polyps in each experimental condition at 0, 4, 24 and 48 h p.a. by maceration, a procedure allowing to dissociate the polyp into fixed single cells than maintain their morphology, and thus enables to quantify each cell type present in the polyp.^[^ ^26^, ^55^ ^]^ A maceration solution (acetic acid, glycerol and H_2_O in a 1:1:13 v/v ratio) was added to living polyps, and incubation was performed at +4 °C overnight. Single cells were fixed through the addition of paraformaldehyde 4%. Dried slides with macerated cells were washed with PBST (PBS 1X‐Tween 0.1%) for l h at room temperature, treated with 2 M HCl for 45 min at room temperature for acid hydrolysis and washed in PBS for 20 min. Blocking was performed through the addition of BSA‐T 1% for 15 min in a wet chamber. Incubations with 1:500 mouse anti‐BrdU (Sigma) in BSA‐T 1% were done for 2 h in a wet chamber. The preparation was then washed in PBS‐T at room temperature and treated with post primary block solution (Novocastra Laboratories Itd) for 30 min. Excess antibody was removed by two 5 min washings with PBS‐T at room temperature. Then Novolinker Polymer solution (Novocastra Laboratories Itd) was added for 30 min. The preparations were washed in PBS‐T and then incubated in DAB (colorimetric reagent and peroxidase substrate) for 5 min. The reaction between the peroxidase linking to the post primary and DAB was blocked by rinsing the slides in water for 5 min. Slides were mounted using Vectashield Antifade Mounting Media with DAPI (Vector Laboratories) and stored at 4 °C. Slides were observed using an inverted microscope (Axiovert 100, Zeiss) equipped with a color digital camera (Olympus DP70) and a fluorescence filter set (BP450‐490/ft510/LP515). Images were acquired by the CellF (Olympus) software. BrdU^+^ cells were detected by their brownish color using a 32x objective and quantified as ratio on a total of ≃600 epithelial cells per slide

### DNA Oxidation

Oxidative DNA damage, namely, 8‐hydroxy‐2 ′‐deoxyguanosine (8‐OHdG) levels, were measured using Epigentek EpiQuik 8‐OHdG DNA Damage Quantification Direct Fluorometric Kit according to the manufacturer's instructions. Briefly, DNA was extracted from 20 *Hydra* in each experimental condition (200 ng) and subjected to 8‐OHdG content analysis. Fluorescence was read at 530_ex_/590_em_ nm using a Tecan Infinite M200 fluorescence mode microplate reader. The calculation was made based on absolute quantification with linear regression function and the 8‐OHdG content in total DNA is presented as %. The results represent the mean ± SD from at least two independent experiments and three technical replicates. Differences between untreated and treated animals were revealed using one‐way ANOVA and Dunnett's multiple comparison test. Two‐tailed paired Student's *t*‐test was also used to compare NP treated animals versus NPs/light treated animals. Statistical significance was evaluated using GraphPad Prism 9. *P*‐values of less than 0.05 were considered significant.

### Levels of 5‐Hydroxymethylcytosine

5‐Hydroxymethylcytosine (5‐hmC) was measured using the MethylFlash Global DNA 5‐Hydroxymethylation ELISA Easy Colorimetric Kit according to the manufacturer's instructions. Briefly, DNA was extracted from 20 *Hydr*a under each experimental condition (40 ng) and subjected to a 5‐hmC content analysis. The absorbance was read at 450 nm using a Tecan Infinite M200 absorbance mode microplate reader. The calculation was made based on absolute quantification with a standard curve and linear regression function, and 5‐hmC content in total DNA is presented as %. The results represent the mean ± SD from at least two independent experiments and three technical replicates. Differences between untreated and treated animals were revealed using one‐way ANOVA and Dunnett's multiple comparison test. The two‐tailed paired Student's *t*‐test was also used to compare NPs treated animals versus NP/light treated animals. Statistical significance was evaluated using GraphPad Prism 9. *P*‐values of less than 0.05 were considered significant.

### Lipid Peroxidation Assay

Lipid peroxidation was determined in homogenized *Hydra* extracts using Lipid Peroxidation (MDA) Assay Kit (Sigma Aldrich) according to manufacturer's protocol using MDA Lysis Buffer. Fluorescence was read at 532_ex_/553_em_ nm using a Tecan Infinite M200 fluorescence mode microplate reader. The level of lipid peroxidation was measured as relative fluorescence units (RFU) and data were normalized to untreated control animals. The results represent the mean ± SD from at least two independent experiments and three technical replicates. Differences between untreated and treated animals were revealed using one‐way ANOVA and Dunnett's multiple comparison test. Two‐tailed paired Student's *t*‐test was also used to compare NPs treated animals versus NP/light treated animals. Statistical significance was evaluated using GraphPad Prism 9. *P*‐values of less than 0.05 were considered significant.

### HaCaT Cells Culture

Immortalised HaCaT cells were cultured in calcium free‐Dulbecco's modified Eagle's medium (DMEM) (catalog number 21969‐035, Gibco Lifetech) supplemented with 1% penicillin and streptomycin, 1% L‐Glutamine 100X (Gibco Lifetech) and 10% Fetal bovine serum (FBS). Cells were maintained in cell culturing sterile flasks, in a humidified atmosphere of 95% air and 5% CO2 at 37 °C in an incubator.

### Cell Migration and Cell Proliferation Assays

HaCaT cells (3 × 10^4^) were seeded into 3 wells cell culture dishes (Ibidi Cat.No: 80 366) with silicone inserts creating in each cell two defined cell‐free gaps. Cells were either treated with NP (100 µg mL^–1^) or untreated and incubated at 37 °C overnight. After washing, the insert was removed and the cells were allowed to close the gap under white illumination (0.124 mW mm^–2^) or in the dark, and imaged in bright field after 8 h and 24 h using an inverted microscope. The relative wound closure was estimated as the covered area observed after 24 h calculated using the following formula: $\frac{fA_{24h}-fA_{0h}}{fA_{0h}}$. To obtain the relative wound closure, the calculated areas were normalized using the control area. Experiments were performed in triplicate and the data was reported as the mean ± SD of three independent biological experiments.

The measure of cell proliferation was obtained by incubating cells, immediately after the removal of the silicone insert, for 4 h with sterile BrdU 10 mM in DMEM. After washing, cells were allowed to close the wound either illuminated for 4 h or not, and after 24 h processed for BrdU^+^ cell detection by immunocytochemistry, as above described for *Hydra*. Slides were observed using an inverted microscope (Axiovert 100, Zeiss). BrdU^+^ HaCaT cells were detected and quantified through a 40x oil immersion objective and images acquired with the software *CellF*.

### Statistical Analysis

No pre‐processing of data was performed. For all cases mean ± SD is represented and *P*‐values of less than 0.05 were considered significant: * = *P* < 0.05; ** = *P* < 0.01; *** = *P* < 0.001.

For regeneration assays: *n* = 90 animals, Chi‐square test was used; for Wnt3 expression levels: *n* = 6, unpaired Stedent *t*‐test; for cell proliferation rates during regeneration: *n* = 600 cells, unpaired Student's *t*‐test. For DNA oxidation: *n* = 6, ANOVA and Dunnett's a posteriori test. For cell migration assay: *n* = 3, unpaired Student's *t*‐test. Graphpad Prism 9 software was used for statistical analysis

## Conflict of Interest

The authors declare no conflict of interest.

## Authors Contribution

G.O. and F.F. contributed equally to this work. G.O. performed in vivo and in vitro experiments with P3HT NPs under the supervision of M.M.; F.F. performed in vivo and in vitro experiments with PCPDTBT NPs; F.G. synthesized and characterized polymer nanoparticles under the supervision of M.R.A.; A.L. and M.W. performed biochemical assays; G.T. carried out BrdU experiments and qRT‐PCR in *Hydra* with PCPDTBT; A.T., M.M., and M.R.A. analyzed data and supervised research. C.T. conceived the project, designed and supervised research, and wrote the manuscript with contributions from all authors.

## Supporting information

Supporting Information

## Data Availability

Data sharing is not applicable to this article as no new data were created or analyzed in this study.
