# Supplementary material for: Optical Control of Tissue Regeneration through Photostimulation of Organic Semiconducting Nanoparticles
Source: Adv Healthc Mater. 2022 Jul 28;11(19):2200366. doi: 10.1002/adhm.202200366 (PMC11469744; doi:10.1002/adhm.202200366)
Supplement: Supplementary file 1 — Supporting Information [file ADHM-11-2200366-s001.pdf]

ADVANCED HEALTHCARE MATERIALS

Supporting Information

for *Adv. Healthcare Mater.*, DOI 10.1002/adhm.202200366

Optical Control of Tissue Regeneration through Photostimulation of Organic Semiconducting Nanoparticles

*Giada Onorato, Federica Fardella, Anna Lewinska, Federico Gobbo, Giuseppina Tommasini, Maciej Wnuk, Angela Tino, Maria Moros, Maria Rosa Antognazza and Claudia Tortiglione**

Supporting Information

Optical Control of Tissue Regeneration Through Photostimulation of Organic Semiconducting Nanoparticles

Giada Onorato[1#$], Federica Fardella[1#], Anna Lewinska[2], Federico Gobbo[3,4], Giuseppina Tommasini[1§], Maciej Wnuk[5], Angela Tino[1], Maria Moros[6], Maria Rosa Antognazza[3], Claudia Tortiglione[1*]

[1]Istituto di Scienze Applicate e Sistemi Intelligenti “E. Caianiello”, Consiglio Nazionale delle Ricerche, Via Campi Flegrei 34, 80078 Pozzuoli, Italy

[2]Department of Biotechnology, Institute of Biology and Biotechnology, Faculty of Biotechnology, University of Rzeszow, Pigonia 1, 35-310 Rzeszow, Poland

[3]Center for Nano Science and Technology @PoliMi, Istituto Italiano di Tecnologia, Via Pascoli 70/3, 20133 Milano, Italy

[4]Politecnico di Milano, Dip. di Fisica, P.zza L. Da Vinci 32, 20133 Milano, Italy

[5]Department of Biology, Faculty of Biotechnology, University of Rzeszow, Pigonia 1, 35-310 Rzeszow, Poland

[6] Instituto de Nanociencia y Materiales de Aragón, C/Mariano Esquillor 15, 50018 Zaragoza, Spain

*email: claudia.tortiglione@cnr.it

Present addresses:

[$] Institute of Biosciences and Bioresources, National Research Council, Via Pietro Castellino 111, Napoli, Italy

[§]Instituto de Nanociencia y Materiales de Aragón, C/Mariano Esquillor 15, 50018 Zaragoza, Spain

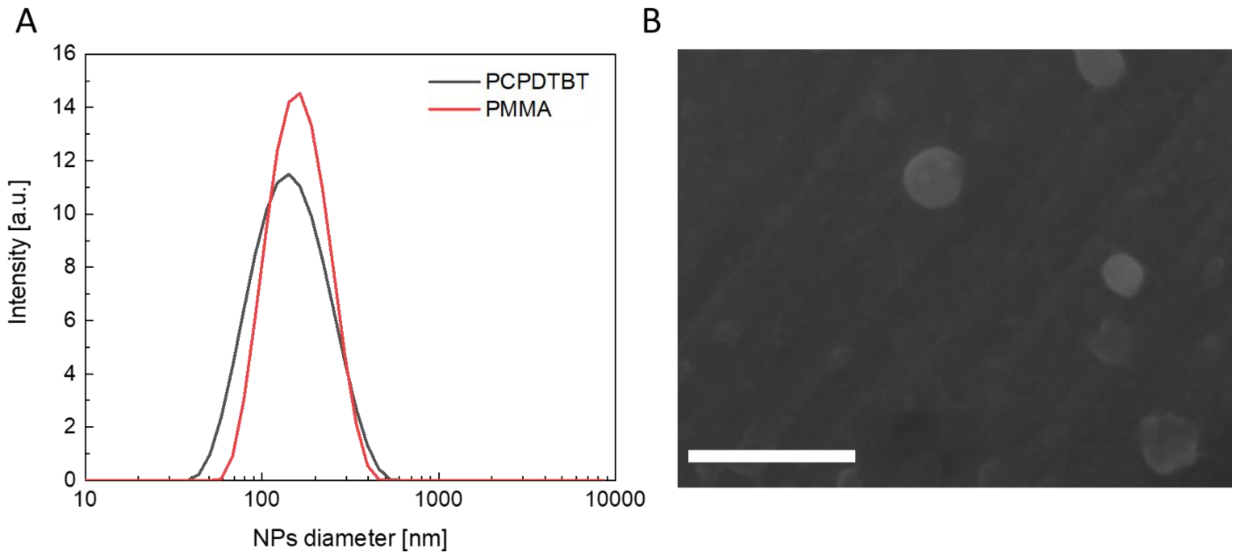

Figure S1- Characterization of PCPDTBT-NPs and PMMA-NPs.

A) Dynamic Light Scattering measurements show average diameter of PCPDTBT-NPs of about 160 nm, comparable to the values obtained for control, optically inert PMMA-NPs (175 nm) and to those of P3HT previously reported (230 nm).[1] B) Scanning Electron Microscope representative image of casted PCPDTBT-NPs. Scale bar, 500 nm.

Toxicological evaluation of PCPDTBT in *Hydra vulgaris*

Hydra is highly sensitive to organic and inorganic compounds and several approaches may be used to determinate the impact of any medium suspended compound on its physiology, i.e., morphology, reproduction rate, regeneration efficiency. We used a morphometric approach as straightforward toxicological endpoint, monitoring the animal morphology in response to increasing dose of PCPDTBT-NP and exposure time. The test employs numerical scores to describe morphological alteration adapted by our group from a previously described method used to assess the toxic effect of organic compounds on *Hydra*.[2] After addition of a test compound to the medium bathing living animals, the morphology is observed and a numerical score assigned to each specimen, from 10 (healthy animal) to zero (disintegrated animal) (Figure S2A). Dose-response curves were determined by exposing group of 30 polyps to PCPDTBT-NP, at doses ranging from 25 to100 μg mL^{-1} in chronic condition and inspecting the animal morphology from 24 h up to 72 h (Figure S2 B-C). No visible effect were detected in these experimental conditions, indicating biosafety of PCPDTBT-NP.

For P3HT-NP the same assay was previously performed, testing doses from 10 μg mL^{-1} up to 400 μg mL^{-1}[1] . The results showed biosafety of the compound, and the population growth curves at doses as high as 1.6 mg mL^{-1} confirmed this effect also on a long term.

To deeper investigate on the biosafety of the two NPs, we performed the assay in presence of light stimulation. We used for both compounds the highest possible dose (400 μg mL^{-1}, dictated by the PCPDTBT synthesis method not allowing to test in the mg range) and performed morphometric assay under light stimulation (0.124mW mm^{-2}). Figure S2 D-E shows that the photostimulation of both NPs do not cause morphological damages up to 72 h.

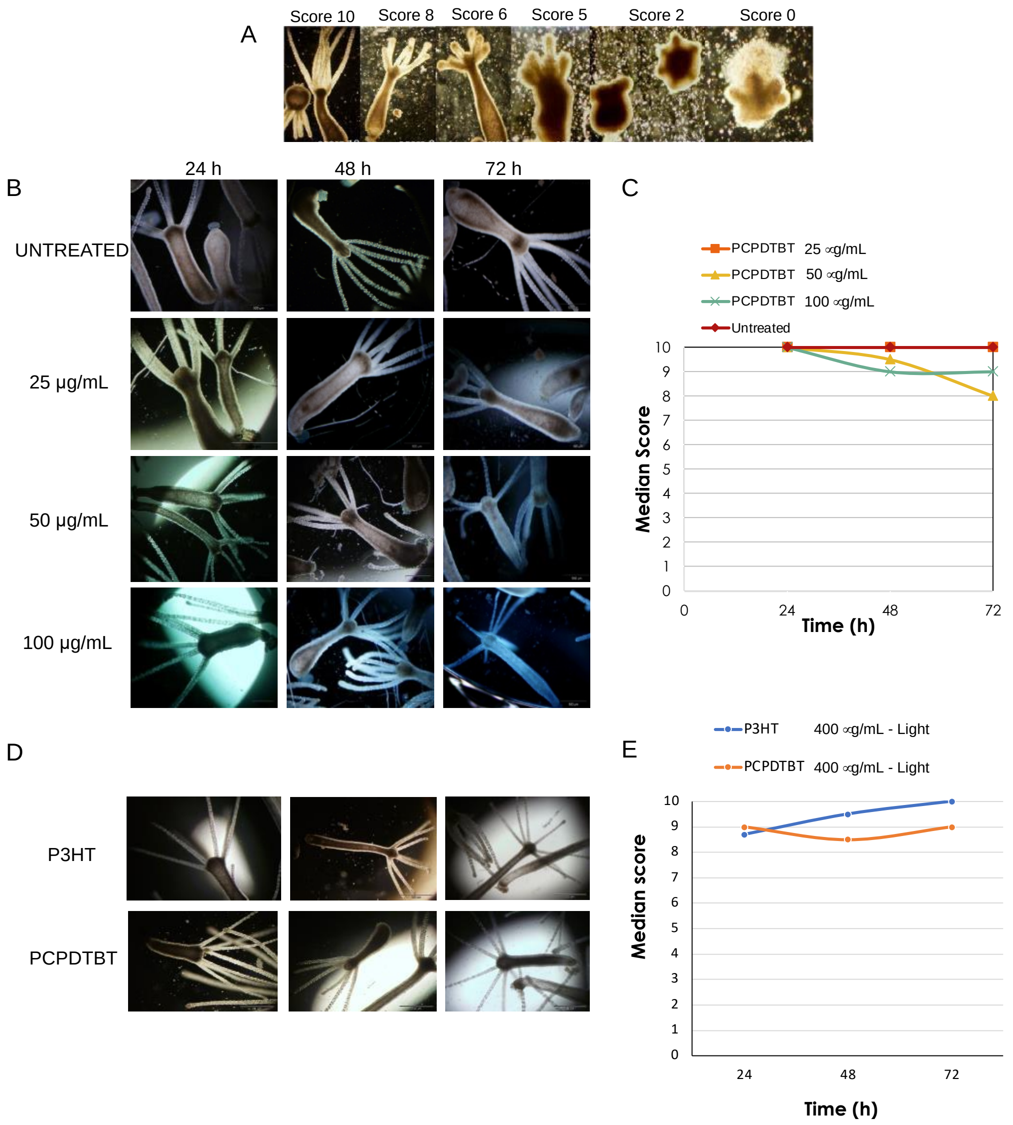

Figure S2. Toxicological evaluation of PCPDTBT-NP in *Hydra vulgaris*

A) Morphometric analysis based on progressive morphological alteration induced by a toxicant on *Hydra*. B) Representative images of polyps exposed to different PCPDTBT NP doses, for increased time periods. C) Dose response curves showing median morphological scores as function of the incubation time, up to 72 h. The morphology of animals was not affected by treatment with PCPDTBT-NP, up to 72 h of continuous incubation with 100 µg mL^{-1}. N=20. D) Representative images and E) quantification of median morphological scores for polyps exposed to PCPDTBT-NP or P3HT-NP at the highest possible concentration (400 µg mL^{-}1) and illuminated with a 12:12 h light: dark regime using a white led light (0.124 mW mm^{-2}). No visible changes in animal morphology were observed. Experiments were performed in triplicate, n=90.

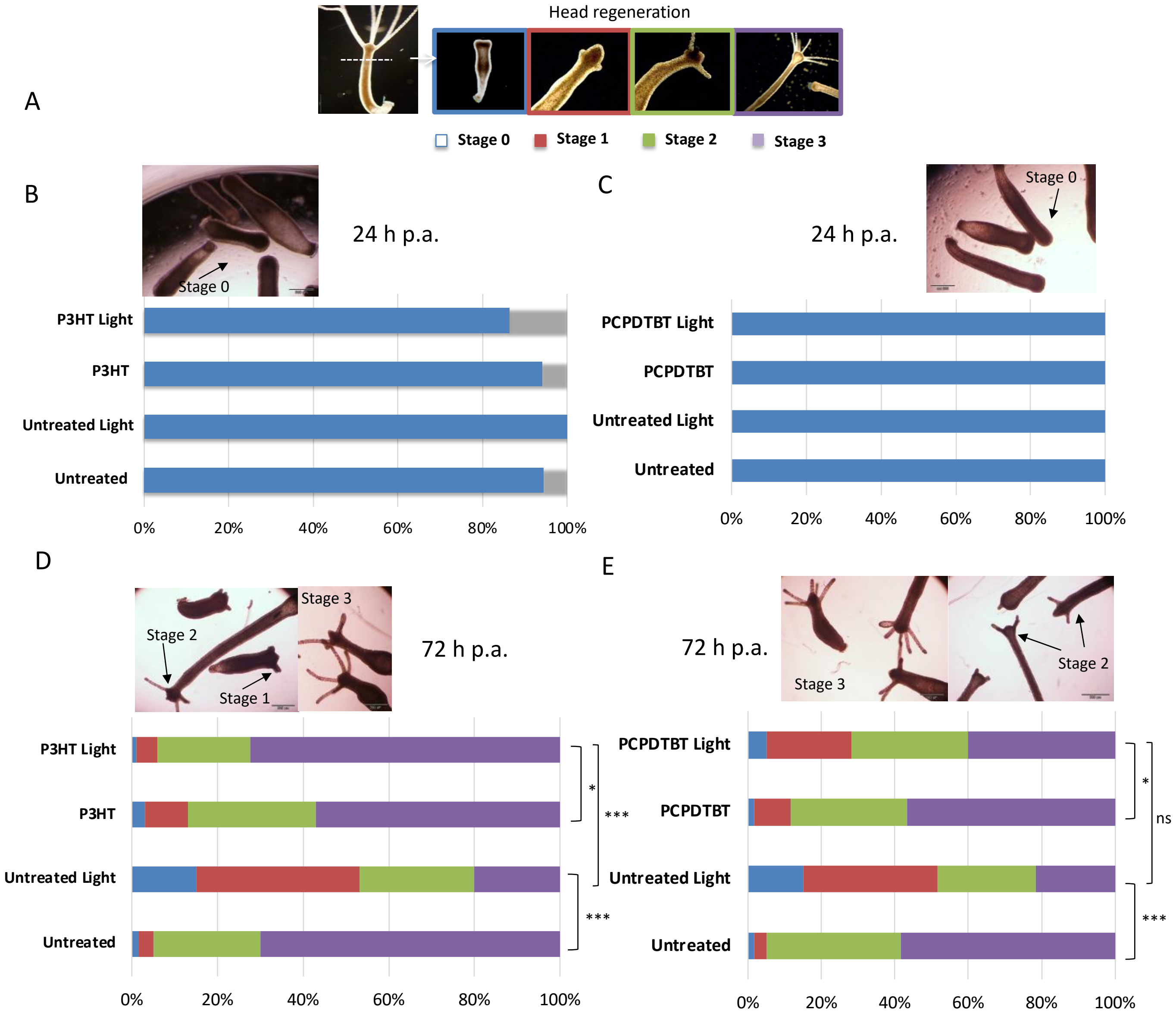

Figure S3. Regeneration efficiency of *Hydra* treated with semiconducting NP at 24 h and 72 h post amputation.

A) Classification of developmental stages of regenerating heads, taken at 24 h intervals. The graphs show the regeneration efficiency (expressed as distribution of developmental stages) of *Hydra* polyps treated with 100 µg ml^{-1} of B) P3HT-NP and C) PCPDTBT-NP, measured 24 h p.a. (B-C) and 72 h p.a. (D-E), while the regeneration efficiencies relative to the 48 h time point are shown in Figure 1C and 1D. Control conditions and light intensity are the same as described in Fig. 1 (0.124 mW mm^{-2}).

The images on the top of each graph show the morphology of the regenerating polyps at the corresponding time p.a., as observed during a typical experiment. At 24 h the wound is completely healed. Only the condition P3HT-NP under illumination presents a low percentage of stage 1 (red

bar). At 72 h p.a. the regeneration is completed in untreated polyps, showing well developed tentacles (stage 2 and 3), and only some aberrant polyp in stage 0 and 1, where the process failed. The distribution of developmental stages mirrors the data collected at 48 h p.a., confirming the enhancing effects played by P3HT-NP and a less extent by PCPDTBT-NP photostimulation.
3 independent biological replicates were performed (n=90). Statistical comparisons were performed using the Chi-square test and the Graphpad Prism 9 software (*P* values: * $P < 0.05$; ** $P < 0.01$; ***$P < 0.001$).

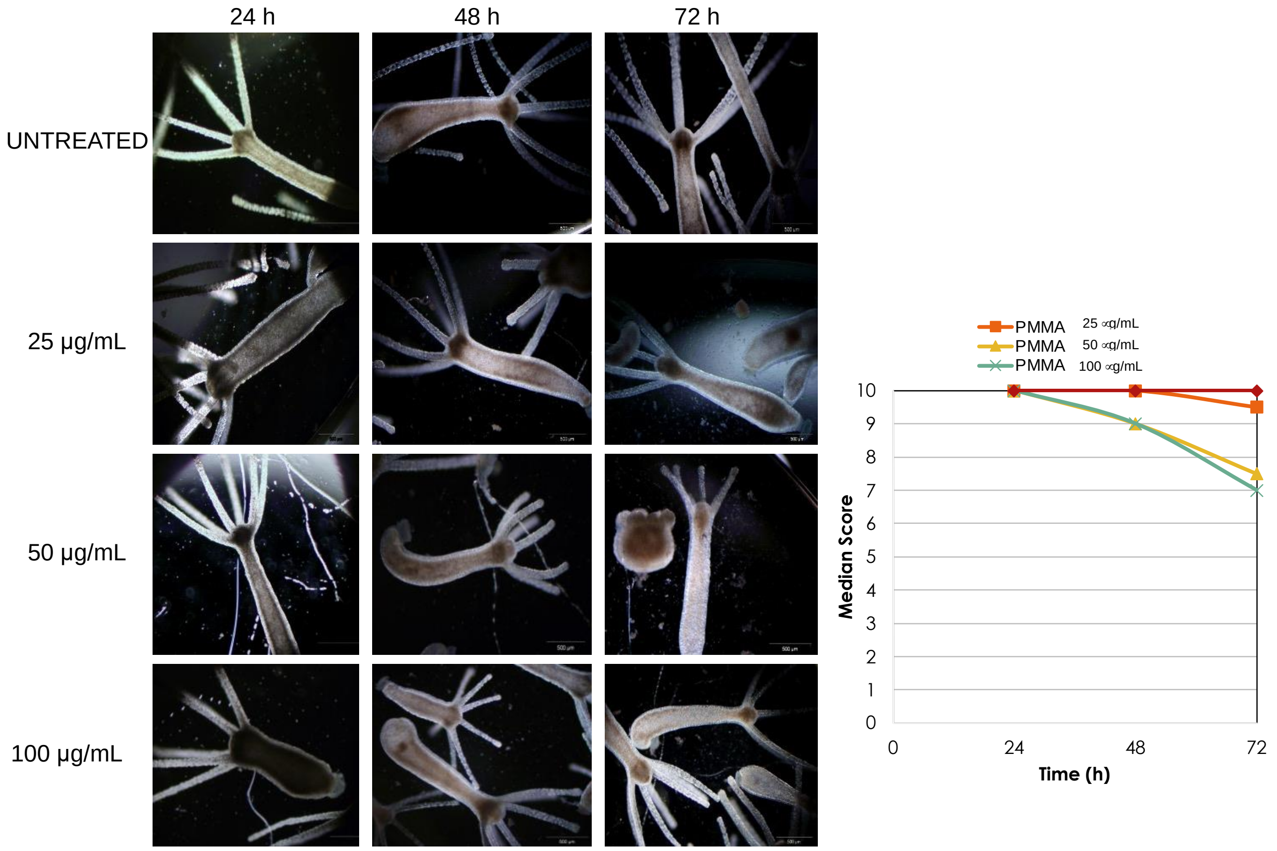

Figure S4. Toxicological evaluation of PMMA-NPs in *Hydra*

Living animals were continuously incubated with PMMA-NP and inspected at various time. The same morphometric assay described in Figure S2 was performed. No signs of morphological alterations were detected up to 72 h of continuous incubation with the highest dose

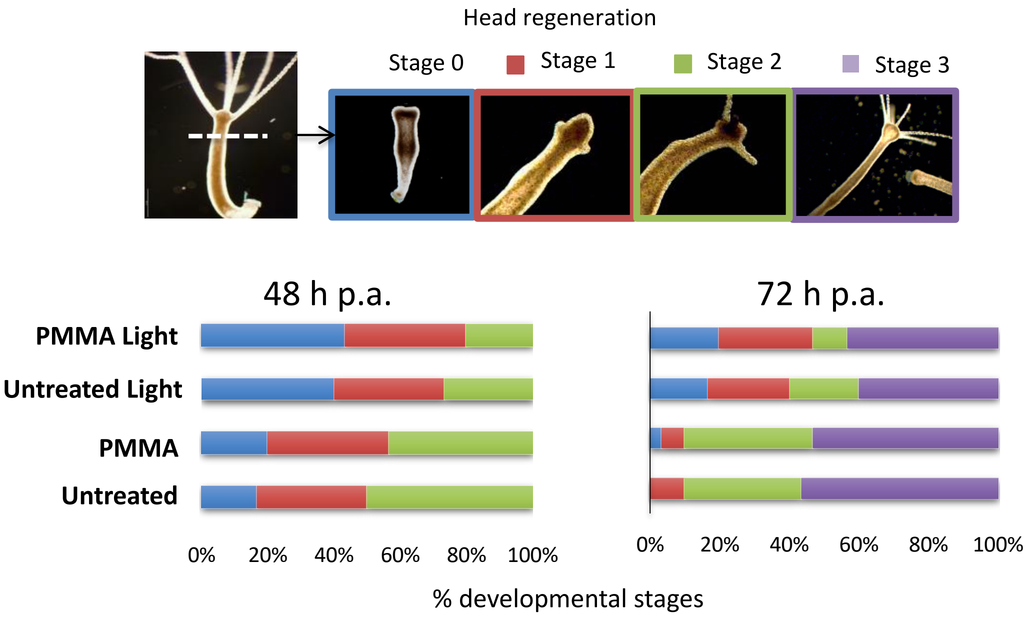

Figure S5. Impact of PMMA-NP on the regenerative capability of *Hydra*

Groups of 20 polyps were treated 24 h with PMMA-NP 100 µg mL^{-1}, and after washing bisected and allowed to regenerate in fresh medium, under white led light (0.124 mW mm^{-2}), or ambient light condition. At the indicated time point post amputation (p.a.) regenerating polyps were inspected to quantify developmental stages, ranging from stage 1 (tentacle bud visible), stage 2 (well-developed tentacle, reaching 2/3 of the mature length) and stage 3 (fully developed tentacles). The histograms show the distribution of the regenerative stages. PMMA treated polyps, either exposed to white light or not, show the same regeneration efficiency of untreated polyps.

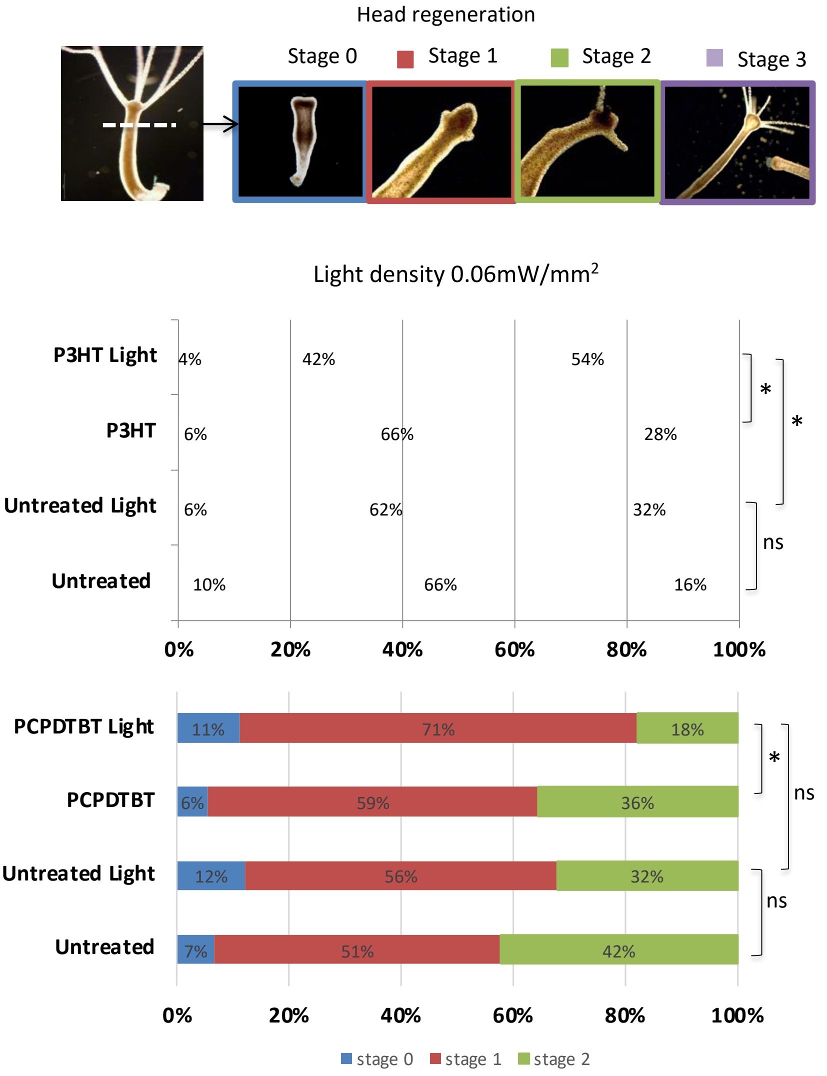

Figure S6. Impact of light intensity on NP photostimulation

Groups of 20 polyps were treated 24 h with the indicated NP 100 µg mL^{-1}, and after washing bisected and allowed to regenerate in fresh medium, under white led light (0.06 mW mm^{-2}), or ambient light condition. 48 h p.a. regenerating polyps were inspected to quantify each developmental stage. While the percentage of stage 2 regenerants was greatly enhanced in animals treated with P3HT-NP and photostimulated, the effect of photostimulation on PCPDTBT-NP treated animals was similar to the other conditions. Each graph represents the average of three biological replicas, each performed with 25 polyps (n= 75). Statistical comparisons were performed using the Chi-square test and the Graphpad Prism 9 software (*P* values: *= *P* <0.05; **= *P* <0.01; ***= *P* <0.001)

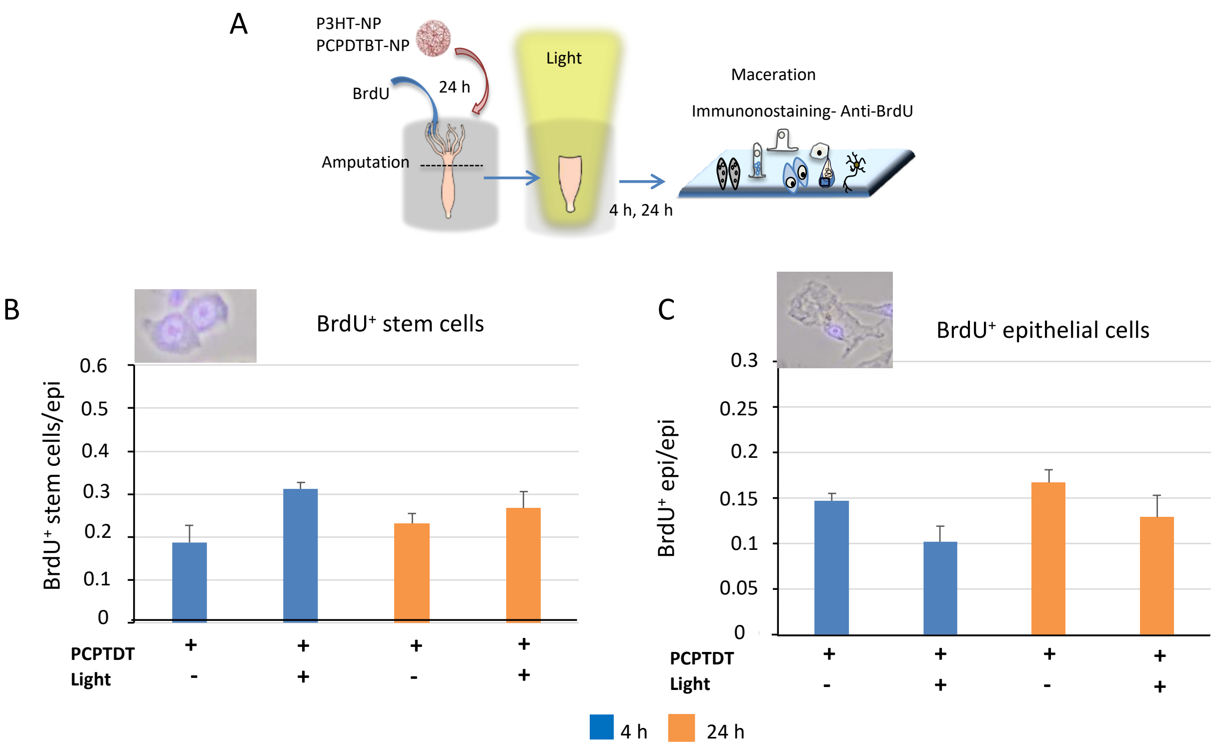

Figure S7. Impact of PCPDTBT-NPs on cell proliferation rates

A) Scheme of the experimental procedure. *Hydra* polyps were treated with PCPDTBT-NP, then pulsed with BrdU, amputated and allowed to regenerate missing heads. At 4 h and 24 h p.a. polyps were macerated and immunostained with anti-BrdU antibody. Relative amount of BrdU$^+$ stem cells B) and epithelial cells C) prepared from treated polyps are indicated as blue and orange bars, corresponding at 4 h and 24 h p.a.: +/- = treated/no light; +/+ = treated/light. For each graph data are presented as means ± SD of three biological replicates (n=600 cells). Statistical comparison using the t-test show no significant difference among the different conditions

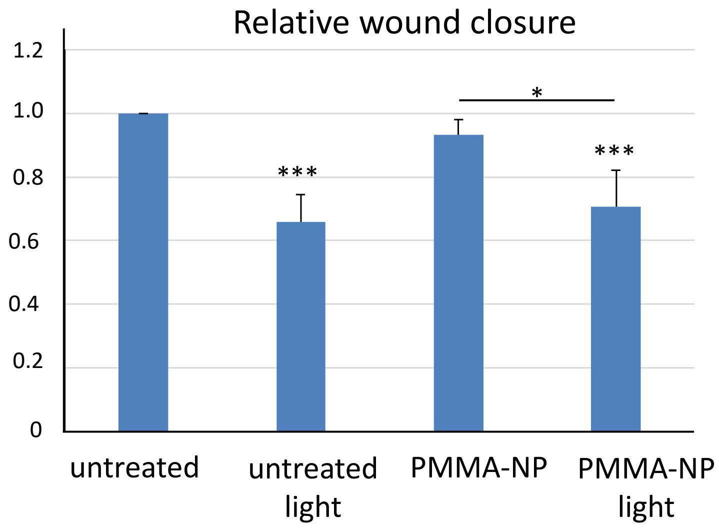

Figure S8. Photostimulation of PMMA-NPs does not affect keratinocyte migration

HaCaT cells either treated with PMMA or untreated were seeded into cell culture dishes. At time zero the insert was removed and cells allowed to close the gap either at light density of 0.124 mW mm^{-2} or at ambient light. Quantification of wound closure, estimated as cell covered area relative to the initial wound area, for each condition, was determined at 24 h after wounding. Experiments were performed in triplicate. Statistical comparisons were performed using unpaired *t* test; *P < 0.05; ***P < 0.001.

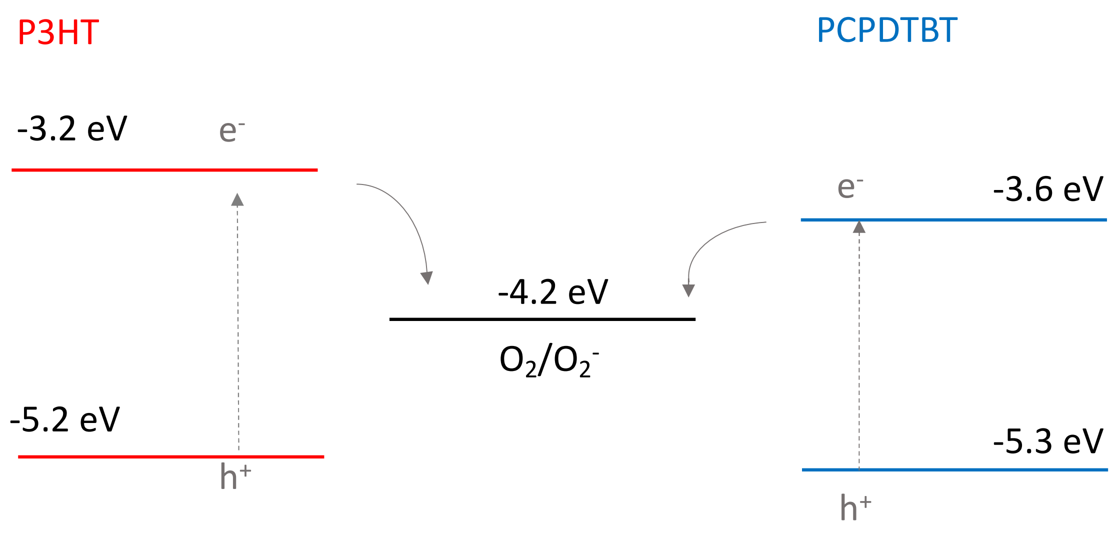

Figure S9. Energetic diagram of P3HT and PCPDTBT conjugated polymers.

Values for HOMO and LUMO levels are from datasheet from the commercial provider [www.ossila.com]. The diagram shows PCPDTBT LUMO level more favourable towards oxygen reduction reactions, and subsequent generation of ROS

Condition	p-value	χ^2value	Significance
Untreated vs P3HT-NP	0.0379	6.548	*
Untreated vs P3HT-NP Light	0.2679	2.634	
Untreated vs Untreated Light	0.0001	60.829	***
Untreated Light vs P3HT-NP	0.0001	78.862	***
Untreated Light vs P3HT-NP Light	0.0001	241.659	***
P3HT-NP vs P3HT-NP Light	0.0004	15.831	***

Table S1. Statistical comparisons relative to the graph of Figure 1C.

Statistical comparisons were performed using the Chi square test, *, $p<0.05$; **, $p<0.01$; ***, $p<0.001$.

Condition	p-value	χ^2 value	Significance
Untreated vs PCPDTBT-NP	0.0086	9.511	**
Untreated vs PCPDTBT-NP light	0.3337	2.195	
Untreated vs Untreated Light	0.0003	16.103	***
Untreated light vs PCPDTBT-NP	0.0001	38.087	***
Untreated light vs PCPDTBT-NP light	0.02	7.820	**
PCPDTBT-NP vs PCPDTBT-NP light	0.0031	11.584	**

Table S2. Statistical comparisons relative to the graph of Figure 1D.

Statistical comparisons were performed using the Chi square test, *, $p<0.05$; **, $p<0.01$; ***, $p<0.001$.

References

[1] C. Tortiglione, M. R. Antognazza, A. Tino, C. Bossio, V. Marchesano, A. Bauduin, M. Zangoli, S. V. Morata, G. Lanzani, *Sci Adv* **2017**, 3, e1601699.

[2] a)M. Allocca, L. Mattera, A. Bauduin, B. Miedziak, M. Moros, L. De Trizio, A. Tino, P. Reiss, A. Ambrosone, C. Tortiglione, *Environmental science & technology* **2019**;
b) A. Ambrosone, L. Mattera, V. Marchesano, A. Quarta, A. S. Susha, A. Tino, A. L. Rogach, C. Tortiglione, *Biomaterials* **2012**, 33, 1991;
c) A. Ambrosone, C. Tortiglione, *Toxicology mechanisms and methods* **2013**, 23, 207;
d) A. Ambrosone, M. Roopin, B. Pelaz, A. M. Abdelmonem, L. M. Ackermann, L. Mattera, M. Allocca, A. Tino, M. Klapper, W. J. Parak, O. Levy, C. Tortiglione, *Nanotoxicology* **2017**, 11, 289;
e) W. Karntanut, D. Pascoe, *Chemosphere* **2002**, 47, 1059; f)O. K. Wilby, J. M. Tesh, *Toxicology in vitro : an international journal published in association with BIBRA* **1990**, 4, 582.
